# Parenting Styles: A Closer Look at a Well-Known Concept

**DOI:** 10.1007/s10826-018-1242-x

**Published:** 2018-09-18

**Authors:** Sofie Kuppens, Eva Ceulemans

**Affiliations:** 1 Erasmus School of Health Policy & Management, Erasmus University Rotterdam, Rotterdam, The Netherlands; 2 Department of Public Health and Primary Care, KU Leuven, Leuven, Belgium; 3 Faculty of Psychology and Educational Sciences, KU Leuven, Leuven, Belgium

**Keywords:** Parenting styles, Cluster analysis, Psychological control, Psychosocial outcomes, School-aged children

## Abstract

Although parenting styles constitute a well-known concept in parenting research, two issues have largely been overlooked in existing studies. In particular, the psychological control dimension has rarely been explicitly modelled and there is limited insight into joint parenting styles that simultaneously characterize maternal and paternal practices and their impact on child development. Using data from a sample of 600 Flemish families raising an 8-to-10 year old child, we identified naturally occurring joint parenting styles. A cluster analysis based on two parenting dimensions (parental support and behavioral control) revealed four congruent parenting styles: an authoritative, positive authoritative, authoritarian and uninvolved parenting style. A subsequent cluster analysis comprising three parenting dimensions (parental support, behavioral and psychological control) yielded similar cluster profiles for the congruent (positive) authoritative and authoritarian parenting styles, while the fourth parenting style was relabeled as a congruent intrusive parenting style. ANOVAs demonstrated that having (positive) authoritative parents associated with the most favorable outcomes, while having authoritarian parents coincided with the least favorable outcomes. Although less pronounced than for the authoritarian style, having intrusive parents also associated with poorer child outcomes. Results demonstrated that accounting for parental psychological control did not yield additional parenting styles, but enhanced our understanding of the pattern among the three parenting dimensions within each parenting style and their association with child outcomes. More similarities than dissimilarities in the parenting of both parents emerged, although adding psychological control slightly enlarged the differences between the scores of mothers and fathers.

Parenting has gained ample research attention from various scientific disciplines. Many theoretical frameworks emphasize that parenting plays a vital role in child development, which has fueled research investigating the impact of parenting on child development for over 75 years. When studying parenting, researchers can take various strategies by considering parenting practices, parenting dimensions or parenting styles. Parenting practices can be defined as directly observable specific behaviors that parents use to socialize their children (Darling and Steinberg 1993). For example, parenting practices intended to promote academic achievement are showing involvement by attending parent–teacher meetings or regular supervision of children’s homework. Other parenting practices pertain to positive reinforcement, discipline, or problem solving.

Rather than focusing on specific parenting practices, other researchers have identified overarching parenting dimensions that reflect similar parenting practices, mostly by modeling the relationships among these parenting practices using factor analytic techniques. There is consensus among scientists about the existence of at least two broad dimensions of parenting, labeled parental support and parental control. Parental support pertains to the affective nature of the parent-child relationship, indicated by showing involvement, acceptance, emotional availability, warmth, and responsivity (Cummings et al. 2000). Support has been related to positive development outcomes in children, such as the prevention of alcohol abuse and deviance (Barnes and Farrell 1992), depression and delinquency (Bean et al. 2006) and externalizing problem behavior (Shaw et al. 1994).

The control dimension has been subdivided into psychological and behavioral control (Barber 1996; Schaefer 1965; Steinberg 1990). Parental behavioral control consists of parenting behavior that attempts to control, manage or regulate child behavior, either through enforcing demands and rules, disciplinary strategies, control of rewards and punishment, or through supervisory functions (Barber 2002; Maccoby 1990; Steinberg 1990). An appropriate amount of behavioral control has been considered to positively affect child development, whereas insufficient (e.g., poor parental monitoring) or excessive behavioral control (e.g., parental physical punishment) has been commonly associated with negative child developmental outcomes, such as deviant behavior, misconduct, depression and anxious affect (e.g., Barnes and Farrell 1992; Coie and Dodge 1998; Galambos et al. 2003; Patterson et al. 1984). While parental behavioral control refers to control over the child’s behavior, parental psychological control pertains to an intrusive type of control in which parents attempt to manipulate children’s thoughts, emotions, and feelings (Barber 1996; Barber et al. 2005). Due to its manipulative and intrusive nature, psychological control has almost exclusively been associated with negative developmental outcomes in children and adolescents, such as depression, antisocial behaviour and relational regression (e.g., Barber and Harmon 2002; Barber et al. 2005; Kuppens et al. 2013). The three parenting dimensions (support, psychological control, and behavioral control) have been labelled conceptually distinct, although they are related to some extent (Barber et al. 2005; Soenens et al. 2012).

Other authors have taken yet a different approach to studying parenting by emphasizing that specific combinations of parenting practices within a parent particularly impact child development rather than separate parenting practices or dimensions (e.g., Baumrind 1991; Maccoby and Martin 1983). Within such a configurational approach, one examines which patterns of parenting practices occur within the same parent and how these patterns—commonly labelled as parenting styles— are related to children’s development. Such parenting styles have the clear advantage of accounting for different parenting practices at the same time within the same person. As such, it comprises a person–centered approach that focuses on configurations within individuals rather than a variable–centered approach that focuses on relationships among variables across individuals as has been used to identify parenting dimensions (Magnusson 1998).

Baumrind (1966, 1967, 1971) is commonly considered a pioneer of research into parenting styles. She introduced a typology with three parenting styles to describe differences in normal parenting behaviors: the authoritarian, authoritative and permissive parenting style. Baumrind (1971) suggested that authoritarian parents try to shape, control, and evaluate their children’s behavior based on the absolute set of standards; whereas permissive parents are warmer and more autonomy granting than controlling. She considered an authoritative parenting style to fall between those two extremes. Later on in the 1980s, Maccoby and Martin (1983) attempted to bridge Baumrind’s typology and parenting dimensions. Based on the combination of two dimensions – demandingness and responsiveness – they defined four parenting styles: authoritative (i.e., high demandingness and high responsiveness); authoritarian (i.e., high demandingness and low responsiveness); indulgent (i.e., low demandingness and high responsiveness); and neglectful (i.e., low demandingness and low responsiveness). These two parenting dimensions are similar, yet not identical to the dimensions ‘parental support’ and ‘parental behavioral control’. Based on Maccoby and Martin’s work, Baumrind (1989, 1991) expanded her typology with a fourth parenting style, namely the ‘neglectful’ parenting style.

Maccoby and Martin (1983) research efforts primarily focused on the configuration of the parenting styles and to a lesser extent on their association with children’s development. Baumrind, in contrast, has also extensively studied the association between parenting styles and child development (1967, 1971, 1989, 1991). This work consistently demonstrated that youth of authoritative parents had the most favorable development outcomes; authoritarian and permissive parenting were associated with negative developmental outcomes; while outcomes for children of neglectful parents were poorest. These aforementioned associations have also been replicated by other researchers. An authoritative parenting style has consistently been associated with positive developmental outcomes in youth, such as psychosocial competence (e.g., maturation, resilience, optimism, self-reliance, social competence, self-esteem) and academic achievement (e.g., Baumrind 1991; Lamborn et al. 1991; Steinberg et al. 1994). Findings regarding permissive/indulgent parenting have been inconsistent yielding associations with internalizing (i.e., anxiety, depression, withdrawn behavior, somatic complaints) and externalizing problem behavior (i.e., school misconduct, delinquency), but also with social skills, self–confidence, self–understanding and active problem coping (e.g., Lamborn et al. 1991; Steinberg et al. 1994; Williams et al. 2009; Wolfradt et al. 2003). An authoritarian parenting style has consistently been associated with negative developmental outcomes, such as aggression, delinquent behaviors, somatic complaints, depersonalisation and anxiety (e.g., Hoeve et al. 2008; Steinberg et al. 1994; Williams et al. 2009; Wolfradt et al. 2003). Children of neglectful parents have shown the least favorable outcomes on multiple domains, such as lacking self-regulation and social responsibility, poor self-reliance and social competence, poor school competence, antisocial behavior and delinquency, anxiety, depression and somatic complaints (e.g., Baumrind 1991; Hoeve et al. 2008; Lamborn et al. 1991; Steinberg et al. 1994).

Baumrind’s typology (1966) was initially determined on theoretical grounds, although with time she did conduct empirical validation research (1967, 1971, 1989, 1991). Nonetheless, the empirical studies always started with parenting styles that were predefined in a prototypical score profile in terms of minimum or maximum limit scores (e.g., scores above or below the median) on the different parenting practices; thus parents were first classified using cut–off scores for these predefined parenting styles and afterwards associations with child developmental outcomes were examined. However, such a confirmatory approach is not preferred to investigate parenting styles types (Mandara 2003) as it does not allow the identification of the naturally occurring typology, because people are actually forced into some predefined category defined on theoretical grounds. To empirically identify typologies in a certain population an exploratory clustering approach is needed (Everitt et al. 2001; Mandara 2003). Such clustering methods entail that persons are assessed on different variables (e.g., parenting practices) and patterns that naturally occur in the data are identified. Persons with a similar score profile are classified in the same cluster and those with distinctly different profile scores are classified into other clusters; with the number of clusters and associated score profiles being unknown a priori. The literature shows that researchers started to adopt such clustering methods in research into parenting styles about 15 to 20 years ago (Aunola et al. 2000; Beato et al. 2016; Brenner andand Fox 1999; Carlson and Tanner 2006; Chaudhuri et al. 2009; Dwairy et al. 2006; Gorman-Smith et al. 2000; Heberle et al. 2015*;* Hoeve et al. 2008; Lee et al. 2006; Mandara and Murray 2002; Martin et al. 2007; McGroder 2000; McKinney and Renk 2008; Meteyer and Perry-Jenkins 2009; Metsäpelto and Pulkkinen 2003; Pereira et al. 2008; Russell et al. 1998; Shucksmith et al. 1995; Tam and Lam 2004; van der Horst and Sleddens 2017; Wolfradt et al. 2003). These studies have generally identified three or four parenting styles that resemble the initial theoretical parenting styles.

Although Baumrind’s typology has greatly influenced parenting research, two issues have largely been overlooked in the existing knowledge. A first issue relates to the psychological control dimension which is currently considered the third parenting dimension. Initially, Baumrind paid little attention to the role of psychological control because her control dimension solely referred to parental socializing practices aimed at integrating the child in the family and society (Darling and Steinberg 1993). In her later work (1971, 1989, 1991), Baumrind did incorporate aspects of psychological control but the confirmatory nature of that research (cf. using predefined clusters) makes it impossible to determine which parenting styles would naturally evolve when psychological control would be taken into account. Empirical studies have also rarely explicitly included parental psychological control when modeling parenting styles. So far, the limited research including psychological control indices (e.g., Pereira et al. 2008; Wolfradt et al. 2003) has mostly identified four parenting styles that match the theoretically distinct styles. Within these parenting styles psychological control coincided with behavioral control levels in the authoritarian parenting style, yet cumulative knowledge remains too limited to draw firm conclusions.

A second issue is that existing research provides little insight into the coexistence of maternal and paternal parenting styles and their joint impact on child development. Although Baumrind included both parents in her studies, she assigned a (pre-defined) parenting style to each one separately. In some studies (1991), data was limited to mothers if both parents were assigned a different parenting style; in others (1971) families were entirely excluded in such instances. Not only Baumrind, but research on parenting styles in general has paid less attention to the impact of joint parenting styles on child development (Martin et al. 2007; McKinney and Renk 2008; Simons and Conger 2007), but has mainly focused on the unique, differential or interaction effects of maternal and paternal parenting styles adopting a variable-oriented perspective (e.g., Beato et al. 2016; Miranda et al. 2016). Children in two-parent households are influenced by the combined practices of both parents (Martin et al. 2007); and some studies have clearly shown that mothers and fathers can differ in their parenting style (Conrade and Ho 2001; McKinney and Renk 2008; Russell et al. 1998). Considering how the parenting styles of both parents cluster together, therefore, aligns more closely with the real experiences of children growing up in two-parent households. Only such an approach can shed light onto possible additive and compensatory effects (Martin et al. 2007). For example, Simons and Conger (2007) found evidence for an additive effect as having two authoritative parents was associated with the most favorable outcomes in adolescents, as well as a compensatory effect where one parent’s authoritative parenting style generally buffered the less effective parenting style of the other parent. Similarly, McKinney and Renk (2008) suggested that in late adolescence perceiving one parent as authoritative while the other parent has a different parenting style, partly buffered for emotional adjustment problems.

Only two studies have simultaneously clustered maternal and paternal practices into joint parenting styles and examined how they are associated with child development (for other approaches, see Martin et al. 2007; Simons and Conger 2007; Steinberg et al. 1994). Meteyer and Perry-Jenkins (2009) modeled the warmth and dysfunctional discipline practices of both parents resulting in three parenting styles that aligned with Baumrind’s typology, namely supportive parents (i.e., similar to Baumrind’s authoritative style), mixed–supportive parents (i.e., mother’s parenting style is similar to Baumrind’s ‘good enough parenting’–style and father’s to Baumrind’s authoritarian style) and non–supportive parents (i.e., similar to Baumrinds’ authoritarian style). Although insightful, this study did not incorporate aspects of psychological control; was limited to early elementary school children (6– to 7– year olds); and was based on a rather small sample size (85 families). McKinney and Renk (2008) identified four joint parenting styles in their cluster analyses using late adolescents’ (18–22 years) reports of authoritative, authoritarian, and permissive parenting: congruent authoritative (i.e., an authoritative parenting style by both parents), congruent authoritarian (i.e., an authoritarian parenting style by both parents), an authoritarian father–authoritative mother combination, and a permissive father–authoritarian mother combination. This study used ratings of parenting styles as input for cluster analysis leaving the role of separate parenting dimensions unclear.

We aimed to extend the existing research on the well-known parenting styles concept by identifying joint parenting styles in an exploratory manner using data on three major parenting dimensions (i.e., support, behavioral control and psychological control) and their associations with child behavioral outcomes in a large sample of mothers and fathers raising elementary school children. In particular, we first examined whether the configuration of exploratory identified parenting styles differed when the – often neglected – psychological control dimension was considered in addition to the support and behavioral control dimensions. Secondly, we identified how parenting practices of mothers and fathers clustered together into joint parenting styles. We were particularly interested in exploring whether similarity or dissimilarity would depict the joint parenting styles. Incongruence could be expected from attachment or gender theories that particularly stress differences between parents’ roles, while assortative or socialization processes could result in highly congruent parenting styles. Thirdly, we associated these joint parenting styles to child behavioral outcomes. For incongruent parenting styles, we particularly examined whether the different parenting styles may buffer each other’s impact on child outcomes. For congruent parenting styles, we looked at additive effects in which parents’ (very) similar styles may reinforce each other’s impact on child outcomes.

## Method

### Participants

Participants were 600 Flemish families with an elementary-school child (301 boys; 299 girls). The children’s age ranged from 8 to 10 years (*M=* 9.27, *SD* = 0.83). For 556 children both parents participated, while for the remaining children only the mother (*n* = 40) or father (*n* = 4) took part in the study. The participating mothers and fathers were on average 38.09 (*SD* = 4.00) and 40.39 years old (*SD* = 4.85), respectively. Most parents received 12 to 15 years of education. The vast majority of children (92%) were of Belgian origin (i.e., children and both parents born in Belgium). The remaining children mostly originated from another European country (*n=* 28); a limited number had an African (*n* = 7), US (*n* = 4), Middle East (*n=* 1), Asian (*n=* 1) or unknown origin (*n* = 7). Most children (84%) lived in traditional two-parent families with married biological parents; others belonged to a blended family (5%), a household with shared custody (2%), or a single-parent household (9%). In this study, we focused on the subsample of families for which both parents consented to participate. Of the initial 556 families, data were available for a final sample of 527 families due to some non-response.

### Procedure

We used data on parenting collected in a Flemish large-scale study on social determinants of child psychosocial functioning including three cohorts: 8–, 9– and 10– year olds. To safeguard representativeness, a two-stage proportional stratified random sample of elementary school children enrolled in mainstream Flemish schools was drawn. In a first stage, 195 Flemish schools were randomly selected taking into account the distribution of schools across the five Flemish provinces and the Brussels region of which 55 schools agreed to participate. In a second stage, 913 children (2nd to 4th grade) were randomly selected within the participating schools. Parents received an introductory letter and consent form via the teachers. Informed consent to participate in the study was obtained for 600 families with both parents participating for 556 children. We used information on parenting practices collected from both parents. The parents received their questionnaires via the teacher during the second trimester and were asked to complete them individually and independently of each other. Given that 583 mothers (98%), and 538 fathers (96%) actually completed the questionnaire, non-response was fairly low.

### Measures

#### Parental behavioral control

Parental behavioral control was operationalized via 19 items of the subscales Rules (8 items; α_mother _= 0.79; α_father _= 0.82)), Discipline (6 items; α_mother_ = 0.78; α_father_ = 0.80) and Harsh Punishment (5 items; α_mother_ = 0.76; α_father_ = 0.80) of the Ghent Parental Behavior Scale (Van Leeuwen and Vermulst 2004). Each item was scored on a 5–point Likert scale from 1 = never true to 5 = always true. The subscale Rules reflects the extent to which parents provide rules for their children’s behavior (e.g., “I teach my child that it is important to behave properly”; “I teach my child to obey rules”). The subscale Discipline pertains to effective punishments after unwanted behavior (e.g., ‘…taking away something nice’; ‘… give him/her a chore for punishment); whereas the subscale Harsh Punishment points towards parental physical punishment when children misbehave (e.g., “I slap my child in the face when he/she misbehaves”; “I spank my child when he/she doesn’t obey rules”; “I shake my child when we have a fight”). We included multiple subscales to represent the multidimensional nature of the behavioral control dimension, as demonstrated by others (Van Leeuwen and Vermulst 2004). In addition, we consider aspects of adequate (i.e., subscales Rules and Discipline) and inadequate behavioral control (i.e., subscale Harsh Punishment) in this study, given the differential association with child outcomes. While the first has been linked to positive child development, the latter has commonly been associated with negative child outcomes. Correlations between maternal and paternal reports were moderate for the subscales Rules (*r* = .31; *p<* .001) and Discipline (*r* = 0.47; *p<* 0.001), but strong for the subscale Harsh Punishment (*r* = 0.52; *p<* 0.001). Within each parent, weak-to-moderate positive correlations were found between the subscales Rules and Discipline (*r*_mother_ = 0.32; *r*_father_ = 0.26; *p<* 0.001); weak positive correlations between the subscales Discipline and Harsh Punishment (*r*_mother_ = 0.22; *r*_father_ = 0.22; *p<* 0.001); and small negative correlations between the subscales Rules and Harsh Punishment (*r*_mother_ = −0.14, *p* = 0.009; *r*_father_ = −0.11; *p=* 0.001).

#### Parental support

Parental support was operationalized by 11 items (1 = never true to 5 = always true) of the subscale Positive Parenting of the Ghent Parental Behavior Scale (Van Leeuwen and Vermulst 2004). This subscale (α_mother _= 0.85; α_father _= 0.88) pertains to parental involvement, positive reinforcement and problem solving (e.g., “I make time to listen to my child, when he/she wants to tell me something”; “I give my child a compliment, hug, or a tap on the shoulder as a reward for good behavior”). Maternal and paternal reports were moderately correlated (*r* = 0.35, *p<* 0.001).

#### Parental psychological control

Parents assessed their own psychologically controlling behavior by means of a Dutch version of the Psychological Control Scale (Barber 1996; Kuppens et al. 2009a) via a 5–point Likert scale from 1 = never true to 5 = always true. This scale (α_mother _= 0.70; α_father = _0.71) included 8 items pertaining to invalidating feelings, constraining verbal expressions, personal attack, and love withdrawal (e.g., “I am less friendly with my child when (s)he doesn’t see things my way”; “If my child has hurt my feelings, I don’t speak to him/her until (s)he pleases me again”; “I change the subject when my child has something to say”). Correlations between maternal and paternal reports were moderate (*r* = 0.32, *p<* 0.001).

#### Child behavioral outcomes

Both parents completed the 20-item Dutch Strengths and Difficulties Questionnaire (SDQ; van Widenfelt et al. 2003) using a 3–point scale in order to assess child psychosocial behavior (0 = not true to 2 = certainly true). Externalizing problems were operationalized via the subscales Conduct Problems (5 items; α_mother_ = .60; α_father_ = 0.61) and Hyperactivity (5 items; α_mother = _0.80; α_father_ = 0.76), while internalizing problems were reflected by the subscale Emotional Symptoms (5 items; α_mother = _0.73; α_father_ = 0.72). We also included the subscale on Prosocial Behavior (5 items; α_mother = _0.67; α_father_ = 0.64). Because high correlations (*r=* 0.54–0.71; *p<* 0.001) between mother and father reports was obtained, an average parental score was created for each subscale.

### Data Analyses

To identify joint parenting styles, we conducted cluster analysis in MATLAB. Cluster analysis is an overarching term for procedures used to identify groups or clusters of individuals based on their scores on a number of variables (Everitt et al. 2001). Greater similarity emerges between individuals of the same cluster (or who lie geometrically closer according to some distance measure) than between individuals from different clusters (Steinly and Brusco 2011). We first ran a cluster analysis based on the four parenting subscales of mothers and fathers (i.e., eight variables as input) that reflect parental support and parental behavioral control to identify joint parenting styles based on these two parenting dimensions (i.e., without considering parental psychological control). To gain insight into the role of parental psychological control in identifying joint parenting styles, we subsequently conducted a cluster analysis on all five parenting subscales of mothers and fathers (i.e., ten variables as input) representing the three parenting dimensions.

We used the conceptual framework of Milligan for a stepwise implementation of cluster analysis (Steinly & Brusco 2011) by (1) determining the observations to be clustered; (2) selecting the variables to be included in the clustering procedure; (3) determining whether and how the selected variables should be standardized; (4) selecting a cluster algorithm and association measure (e.g., a distance measure); (5) determining the number of clusters; and (6) validating clustering (i.e., interpretation, testing, and replication). During steps 1 through 3, we performed analyses on the sum scores of the different parenting subscales which were standardized to give each variable equal weight in the analysis. In step 4, we chose Mac Queens K–means cluster algorithm which aims to identify *K*–clusters with the largest possible between–cluster differences and the smallest possible within–cluster differences (Everitt et al. 2001), while the value of *K* is specified by the user. K-means consists of a reallocation procedure by which persons, starting from an initial random or rational clustering, are reallocated in clusters as long as this yields a decrease in the loss function (i.e., sum of squared Euclidean distance from the corresponding cluster mean). Because the resulting clustering strongly depends on the initial clustering (Steinley 2003), we used 1000 random starts and retained the clustering with the lowest loss function value. To determine the optimal number of clusters in step 5, or in other words to define the value of *K*, we used the CHull procedure (Ceulemans and Kiers 2006; Wilderjans et al. 2013). CHull is an automated model selection procedure that scans a complexity versus fit plot to find the model with the best complexity versus fit balance. Applied to K-means clustering, this means that we look for the model after which allowing for additional clusters does not substantially decrease the loss function. To interpret the resulting clusters (step 6), we visually inspected the pattern emerging in the cluster profile plots. When comparing the cluster-specific profile scores between parents, we focused on the position of the corresponding profile scores compared to zero (i.e., the standardized mean of the sample) and differences in its substantial interpretation. For example, the terms above and below average mean that a parent scores higher or lower than the standardized mean of the sample.

To assess the validity of the empirically identified joint parenting styles representing all parenting dimensions, we examined their association with child behavioral outcomes via four analyses of variance (ANOVA) using SPSS Version 23 with the SDQ-subscales as dependent variables and the identified joint parenting styles based on the three parenting dimensions as the independent variable. Analyses of residuals did not reveal meaningful violations of model assumptions.

## Results

In the following sections, the empirically identified joint parenting styles based on the four subscales reflecting the two parenting dimensions ‘support’ and ‘behavioral control’ are first presented; followed by the results of analyses also considering ‘parental psychological control’ as input behavior. We end with linking the identified joint parenting styles based on three parenting dimensions to child behavioral outcomes.

### Clusters with Two Parenting Dimensions

**Fig. 1:**
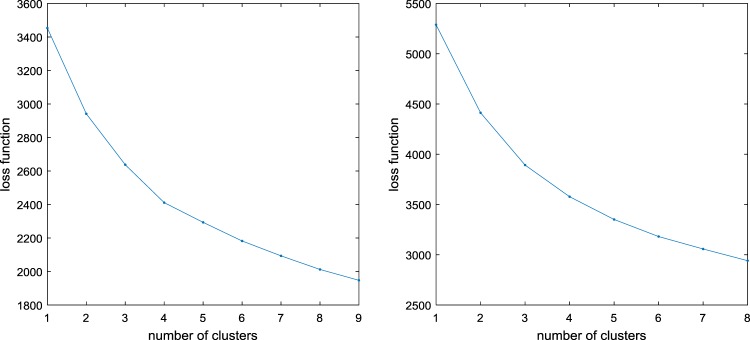
Number of clusters vs. loss function plots for the cluster analyses based on the two parenting dimensions (left) and on the three parenting dimensions (right)

In a first step, we conducted a *K*–means cluster analysis on the maternal and paternal ratings only using the four parental support and behavioral support subscales for each parent (i.e., eight variables) as input, representing the two parenting dimensions. The analysis was conducted for 1 to 8 clusters each with a 1000 random starts. The corresponding number of clusters versus loss function plot is shown in Fig. 1. Applying the CHull procedure to this plot pointed towards a solution with four clusters.

**Fig. 2:**
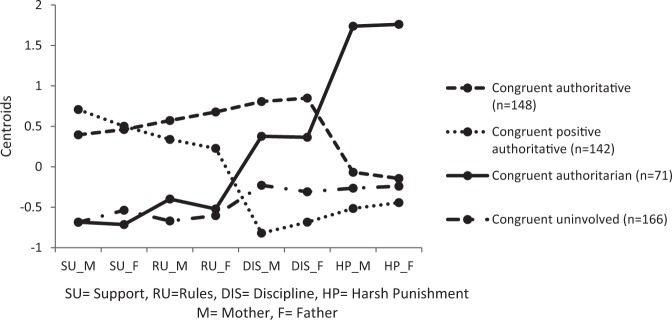
Cluster profiles of the analysis based on two parenting dimensions

Parents belonging to the first cluster (Fig. 2) scored above average on positive parenting, rules and discipline; and scored below average on harsh punishment. A visual inspection of the cluster plot did not reveal notable differences between mothers and fathers. These parents show warmth and involvement in their interaction with their child, but at the same time set clear rules and expectations for children’s behavior. They also discipline the child’s undesirable behavior, but rarely use strict physical punishment when doing so. Because these parents demonstrate elevated support and (adequate) behavioral control levels, we labeled this parenting style as the *congruent authoritative* parenting style.

Parents belonging to the second cluster (Fig. 2) also scored above average on positive parenting and rules, but clearly below average on effective (subscale Discipline) and harsh disciplining (subscale Harsh Punishment). Based on a visual inspection, levels of positive parenting and providing rules of mothers seemed somewhat higher, while effective discipline was somewhat lower compared to fathers, but the substantive interpretation was similar across parents. These parents show warmth and involvement in their parenting while also setting clear rules for children’s behavior, yet they hardly discipline their child in any manner after showing unwanted behavior. Because these parents showed elevated support levels combined with aspects of behavioral control that focus on promoting desired behavior (instead of discouraging unwanted behavior), we labeled this cluster as the *congruent positive authoritative* parenting style.

The third cluster (Fig. 2) included parents who scored clearly above average on harsh punishment, above average on discipline, and below average on positive parenting and rules; without any notable visual differences between mothers and fathers. These parents are therefore less warm and involved in the relationship with their child. Their parenting is particularly characterized by strict physical punishment following unwanted behavior, without setting clear rules for their children’s behavior. This cluster reflected the *congruent authoritarian parenting style*.

A fourth cluster (Fig. 2) was identified that yielded below average scores for both parents on all subscales; without salient visual differences between mothers and fathers. These parents do not show marked warmth and involvement with their child, and also do not prominently provide rules or discipline unwanted behavior. Because these parents demonstrated below average scores on both dimensions, we labeled this cluster as a *congruent uninvolved* parenting style.

### Clusters with Three Parenting Dimensions

**Fig. 3:**
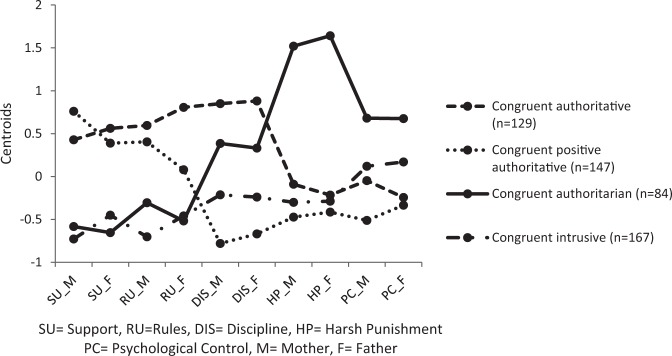
Cluster profiles of the analysis based on three parenting dimensions

In a second step, we performed the same *K*–means cluster analysis, but now psychological control was included as a third parenting dimension. The analysis was again conducted for 1 to 8 clusters each time using 1000 random starts. Applying the CHull procedure to the number of clusters versus loss function plot (Fig. 1) pointed toward a solution with 2 or 3 clusters. However, to enable comparisons between the cluster solution based on the two parenting dimensions, we again selected the solution with four clusters of which the cluster profiles are visualized in Fig. 3.

When comparing both cluster solutions, a remarkable similarity in the cluster profiles was observed with the cluster scores on parental psychological control for the *congruent authoritative, congruent positive authoritative* and *congruent authoritarian* parenting styles covarying with scores on harsh punishment. These three clusters could thus be interpreted and labeled in a similar manner as earlier. For the *congruent uninvolved* parenting styles, the pattern for parental support and behavioral control remained fairly unchanged, but both showed slightly above-average psychological control scores. It seems that these parents are thus less supportive and behavioral controlling, yet showing somewhat elevated levels of psychologically intrusive practices. As such, we relabeled the congruent uninvolved cluster as a *congruent intrusive* parenting style. Adding the psychological control dimension slightly enlarged the differences between the scores of mothers and fathers within each parenting style, but the substantive interpretation remained similar across parents

Given the substantial similarity in emerging parenting styles after including two or three parenting dimensions, we computed the agreement in classification of the corresponding parents. Analyses revealed that parents were generally assigned to the same parenting style if psychological control was taken into account, (Cramer’s *V* = .87). Note that the agreement was substantial regardless of the retained number of clusters (2 clusters: *V=* .77; 3 clusters: *V=* .86; 5 clusters: *V=* .83; 6 clusters: *V=* .69; 7 clusters: *V=* .68; 8 clusters: *V=* .65).

### Parenting Styles and Child Behavioral Outcomes

**Fig. 4:**
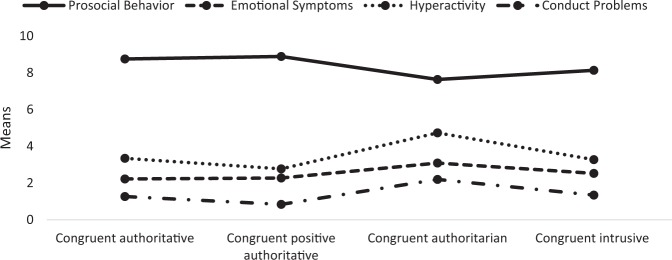
Mean subscale scores on child behavioral outcomes per parenting style

The four joint parenting styles were associated to significantly different behavioral outcomes: Prosocial Behavior [*F*(3, 520) = 20.15, *p<* 0.001, *R*^*2*^= 0.10]; Hyperactivity [*F*(3, 520) = 12.98, *p<* 0.001, *R*^*2*^*=* 0.07]; Emotional Symptoms [*F*(3, 520) = 3.77, *p=* .011, *R*^*2*^= 0.02]; and Conduct Problems [*F*(3, 520) = 20.15, *p<* 0.001, *R*^*2*^= 0.10]. The mean subscale score per joint parenting style are presented in Fig. 4. To gain more insight into the nature of the differences, pairwise contrasts (Tukey–Kramer) were computed for each ANOVA.

For each child behavioral outcome, a significant difference (*p* < 0.05) was established between the *congruent authoritarian* parenting style and at least one other parenting style. Children of authoritarian parents demonstrated more negative (i.e., hyperactivity, conduct problems, emotional symptoms) and less positive (i.e., prosocial behavior) child outcomes compared to children whose parents belonged to another parenting style. For conduct problems, the associated standardized mean difference involving authoritarian parents was most pronounced compared to positive authoritative parents (*d=* 1.06, *p<* 0.001), whereas a medium difference (range *d=* 0.67 – 0.73, *p<* .001) with the authoritative and intrusive parenting styles was found. Similarly, for hyperactivity standardized mean differences involving authoritarian parents were large (*d=* 0.85, *p<* 0.001) compared to positive authoritative parents; and medium (range *d=* 0.60 – 0.63, *p<* 0.001) compared to authoritative and intrusive parents. Standardized mean differences involving authoritarian parents were large (range *d=* 0.83–0.93, *p<* 0.001) for prosocial behavior, but only a small difference (*d=* 0.37, *p=* 0.031) with the intrusive parenting style emerged. Standardized mean differences for emotional symptoms between the authoritarian parenting style were small in magnitude (range *d=* 0.40 – 0.43, *p<* 0.05), except for a non-significant (*d=* 0.28, *p=* 0.159) difference with the intrusive parenting style.

In addition, the *congruent positive authoritative* parenting style yielded significantly lower conduct problem levels in children (range *d=* 0.33 – 0.39, *p<* 0.05) compared to authoritative and intrusive parents. In contrast, significantly less prosocial child behavior (range *d=* 0.46–0.56, *p≤* 0.001) was found for the *congruent intrusive* parenting style compared to (positive) authoritative parents.

## Discussion

With this study, we aimed to add to the parenting styles literature by identifying empirically derived joint parenting styles based on data regarding the three major parenting dimensions as perceived by both mothers and fathers raising elementary school children. These resulting joint parenting styles were subsequently associated with child behavioral outcomes. As highlighted in the introduction, the commonly used parenting typologies have a theoretical underpinning, although empirical studies have generally identified three or four similar parenting styles. Our empirically derived parenting styles based on the two parenting dimensions Support and Behavioral Control bear resemblance to the initial authoritative, authoritarian, and neglectful parenting styles, yet some differences also emerged.

The authoritative parenting style was further broken down into a disciplinary and non-disciplinary subtype. Similarly, although differences between parents within each parenting style were minor, they were more pronounced for the non-disciplinary than for the disciplinary control strategies. These findings highlight that all parenting practices aimed at controlling, managing or regulating child behavior are not necessarily simultaneously used by the same parent, suggesting that considering a variety of parenting practices is crucial to identifying naturally occurring parenting substyles. Some parents seem to provide clear rules, guidelines and expectations for child behavior, but hardly have deviant child behavior followed by an effective disciplinary strategy. One subgroup appears to reflect parents that mostly adopt positive parenting practices (i.e., high support, high rule setting), whereas another subgroup uses a combination of positive (i.e., high support, high rule setting) and negative (i.e., high effective discipline) parenting practices. The latter closely resembles the authoritative parenting style as originally defined (Baumrind 1966, 1967, 1971), while the former clustering aligns more with a second–order positive dimension obtained in research adopting a variable–oriented approach (Van Leeuwen et al. 2004).

In this study, the positive dimension tapped into parenting practices such as parental involvement, positive reinforcement, rule setting, and autonomy–stimulating behavior, while the negative dimensions pertained to negatively controlling efforts such as effective discipline, ignoring or harsh punishment following children’s unwanted behavior. In the uninvolved parenting style, parenting practices bear a resemblance to the neglectful parenting style given the below average scores on all subscales suggesting that parents show less warmth, place fewer restraints on and display little monitoring of children’s behavior. However, we did not identify extreme low scores on parenting dimensions that would suggest a truly neglectful parenting style as originally defined; thus an uninvolved parenting style seems a more appropriate label. Although parent self-reports could overestimate scores of positive parenting and underestimate scores of negative parenting due to social desirability bias, it should be noted that a previous study using adolescent reports also did not find extreme scores for the parenting style clusters (McKinney and Renk 2008).

We were not able to empirically identify the originally proposed permissive parenting style reflecting parents that are very loving, warm and involved (high support), yet have relatively few rules for children’s behavior and hardly discipline (low behavioral control). This finding diverges from some previous empirical studies in which the latter parenting style did emerge using an a theoretical (Aunola et al. 2000; Carlson and Tanner 2006; Shucksmith et al. 1995; Wolfradt et al. 2003) or empirical clustering approach (McKinney and Renk 2008). Our operationalization of the support dimension via the positive parenting subscale of the Ghent Parental Behavior Scale could underlie this divergent finding, because the subscale does not only pertain to warm and responsive parenting practices, but also includes items on problem solving. In contrast to other studies tapping only into warmth and responsiveness, lower scores on solving problems together with the child can attenuate overall scores on parental support. As a result, the pronounced scores on parental support which typify a permissive parenting style may have been somewhat masked in the present study. Alternatively, the parent self-reports may not accurately reflect their actual parenting practices due to a social desirability bias, hampering the identification of the permissive parenting style.

Regarding the role of psychological control in empirically deriving parenting styles, cluster analyses revealed a very similar configuration with four parenting styles when parental psychological control was taken into account. Thus, its addition did not lead to the identification of additional parenting styles, but the third parenting dimension did enhance our understanding. Results clearly pointed toward a substantial overlap between parental psychological control and parental harsh punishment for the congruent authoritarian, authoritative and positive authoritative parenting styles. This finding coincides with research suggesting that inadequate behavior control (e.g., physical punishment) and psychological control by parents are correlated, whereas parental psychological control and adequate behavioral control are considered orthogonal dimensions (Barber 1996; Gray andand Steinberg 1999; Steinberg 1990). For example, Pettit et al. (2001) found that parental psychological control was preceded in adolescence by harsh, restrictive disciplinary parenting during childhood. Barber and Harmon (2002) have further argued that parental psychological control may be a marker of a hostile and dysfunctional parent – child relationship, including the use of harsh disciplinary parenting practices.

For the congruent uninvolved parenting style, including parental psychological control actually led to an improved understanding of the previously considered uninvolved parents. As it turned out these parents did use psychologically controlling strategies to some extent, regardless of their lower levels on the other parenting dimension. This pattern could mean that in the parents–child relationship these parents are not so much concerned with the child and their behavior, but with manipulating children’s thoughts, emotions, and feelings to fit their own. It is commonly recognized that by using psychologically controlling strategies, parents intrude into children’s ‘psychological world’, exert parental authority over the children’s own life, and intervene in the individuation process (Barber and Xia 2013; Steinberg 2005). A recent study by Zhang et al. (2015) also demonstrated that parental psychological control indeed positively correlated with parent–centered intentions, implying that parents intend to satisfy their own needs by applying controlling behaviors with their children.

Several theories point towards differences in parenting between mother and father (McKinney and Renk 2008). For example, psychoanalytic theory argues that mothers are children’s primary attachment figure whereas a greater distance between fathers and their children occurs; the gender and role theory link differences in child rearing to male and female characteristics (e.g., expressiveness and instrumentality) with the traditional mother role as caring figures and fathers taking on the role of authority figure and family provider. The literature also indicates that differences in parenting between mothers and fathers may arise if one parent wants to compensate for the other parent (Meteyer and Perry-Jenkins 2009; Simons and Conger 2007). Nonetheless, our results revealed more similarities than dissimilarities in the parenting styles of both parents, despite small-to-moderate correlations between mother and father reports. These similarities may reflect an assortative process when choosing a partner, meaning that people tend to look for a partner with similar characteristics (Botwin et al. 1997; Buss 1984, 1985; Larsen and Buss 2010). Similarity in parenting could also result from socialization processes (Simons and Conger 2007); through a process of mutual influence or reciprocity partners gradually form similar views and beliefs on parenting. The slight differences that emerged pertained particularly to a dissimilar position on positive parenting and rule setting. Although less pronounced, this finding aligns with the study by Meteyer and Perry-Jenkins (2009) that yielded congruent parenting styles for mothers and fathers of 7-year old children, except for a dissimilar position on self-reported parental warmth. Another study using adolescent reports of parenting (McKinney and Renk 2008) found more pronounced sex differences. Perhaps sex differences in parenting styles become more apparent as children grow older or when children’s perspectives are considered.

Results on associations between the joint parenting styles and child behavioral outcomes indicated that children of two authoritarian parents showed the poorest behavioral outcomes. These children were perceived as showing significantly more internalizing and externalizing problem behavior and less prosocial behavior compared to children of parents adopting other parenting styles. In contrast, children of two positive authoritative parents demonstrated the lowest levels of conduct problems. These findings could suggest an additive effect in which the impact of similar parenting styles is reinforced as having two authoritarian and two positive authoritative parents was associated with the least and most favorable child behavioral outcomes, respectively.

The obtained associations between parenting styles and child behavioral outcomes partially align with previous research. Firstly, it has repeatedly been demonstrated that an authoritative parenting style coincides most with positive developmental outcomes in children (e.g., Aunola et al. 2000; Baumrind 1967, 1971, 1989, 1991, Darling and Steinberg 1993; Dornbusch et al. 1987; Lamborn et al. 1991; Querido et al. 2002; Shucksmith et al. 1995; Steinberg et al. 1994; Steinberg et al. 1992). Our findings confirm this pattern for the children having parents who employ an authoritative parenting style, but children with parents both using a positive authoritative parenting style even showed less conduct problems. This finding could point towards the value of rule setting – in contrast to disciplinary strategies – in preventing behavioral problems. However, as parenting is a reciprocal process with children and parents mutually influencing each other, it is equally likely that parents show less disciplinary strategies simply because their children pose fewer behavior problems as demonstrated by others (Kerr et al. 2012; Kuppens et al. 2009b; Laird et al. 2003).

Secondly, previous research has repeatedly linked an authoritarian parenting style with externalizing and internalizing behavior problems in children (e.g., Hoeve et al. 2008; Lamborn et al. 1991; Steinberg et al. 1994; Williams et al. 2009; Wolfradt et al. 2003). The present findings extend this body of research, although the association was most pronounced for externalizing behavior problems which may be due to children’s age (8 to 10 year olds). In younger children, having authoritarian parents may be more strongly associated with externalizing problem behavior, whereas the association with internalizing problems only emerges as children grow older. The shift in the nature of behavior problems as children age has been linked to the physical, cognitive and social maturation of children and the associated changes in social demands and expectations.

Thirdly, the neglectful parenting style has been associated with the poorest developmental outcomes in children (Baumrind 1991; Lamborn et al. 1991; Mandara and Murray 2002; Shucksmith et al. 1995; Steinberg et al. 1994). As this parenting style did not emerge in the present study, we were not able to model its association with child outcomes. Even children having parents who were less involved, but intrusive, were doing better than children having authoritarian parents. Findings did reveal that prosocial behavior and conduct problems were significantly lower for children having parents who adopted an intrusive parenting style compared to children of (positive) authoritarian parents. This findings coincides with a growing body of evidence on the deleterious of impact of psychologically controlling parenting in children and adolescents adopting a variable approach (Barber et al. 2005; Kuppens et al. 2013; Soenens et al. 2012), but likewise extends this evidence-base with person-oriented findings on the impact of an intrusive parenting style on child development.

### Limitations and Future Research

Although the present study has several merits, it falls short in that only parent self-reports were used to assess parenting and child behavioral outcomes; children’s perspective on their parenting practices may be quite different. For example, Smetana (1995) found that adolescents perceived their parents as being more permissive and authoritarian compared to parents’ own view on the matter, whereas parents perceived themselves as being more authoritative than their adolescent children. Although a significant convergence between child and parent reports on parenting dimensions has been established in elementary school (Kuppens et al. 2009a), future research should explicitly take a multiple informant approach when identifying parenting styles as informant perspectives on parenting styles in this age period may differ. In a related vein, multiple informant assessments of child behavioral problems have been shown to be context–specific with differences occurring according to the context (e.g., home, school) that forms the basis for informant’s assessment (Achenbach et al. 1987). Involving informants other than parents in the assessment of child behavioral outcomes therefore seems particularly interesting in future research on parenting styles.

Furthermore, inspecting a normally developing sample generally results into a low occurrence of inadequate parenting practices and child behavioral problems. Studying parenting styles in a clinical sample could certainly supplement this view because more variation in parenting practices may yield more or different parenting styles. Hoeve et al. (2008) have conducted one of the few studies using a sample of children with a high or low risk of antisocial and behavioral problems; and they were able to identify a neglectful parenting style. In addition, the role of parental psychological control in identifying parenting styles may be more pronounced in a clinical sample; an issue that to date remains unresolved.

The present sample closely resembled the population distribution with regard to family composition and paternal educational level, but it was rather homogeneous for ethnicity and mothers were more highly educated. As such, the present findings may not generalize to minority groups or families with less educated mothers; an issue that should be resolved by future studies. For example, previous research has demonstrated that harsh punishment and psychological control are more common among lower SES parents (e.g., Eamon 2001; El‐Sheikh et al. 2010) and that Caucasian caregivers were more prevalent in an authoritative parenting style cluster (van der Horst and Sleddens 2017). The present study clearly complements the scarce body of research on naturally occurring joint parenting styles conducted in US samples, but additional research is needed to replicate these findings. Moreover, as parenting occurs within a cultural belief system that influences attitudes towards particular parenting practices (Durrant et al. 2003), cross-cultural research could further clarify the role of culture in identifying naturally occurring (joint) parenting styles incorporating three parenting dimensions. Finally, the cross-sectional associations among joint parenting styles and child outcomes should be complemented by longitudinal research to gain more insight into the directionality of these associations. Longitudinal research covering the entire childhood and adolescence period could also increase our understanding of age-of-child and sex-of parent differences in naturally occurring parenting styles.

Despite these limitations, this study adds to the literature by further empirically validating well-known parenting styles and by increasing our understanding of the role of parental psychological control and joint parenting. The overlap between harsh punishment and parental psychological control in congruent parenting styles and its unique role in the uninvolved parenting style suggests that this intrusive parenting dimension should be routinely considered in practice settings. We also found that adequate behavior controlling practices may be particularly interesting in preventing behavioral problems; and that not only an authoritarian but also a (psychologically) intrusive parenting style can impede upon child development.
